# Supplementary material for: A cell atlas of the developing human outflow tract of the heart and its adult aortic valve derivatives
Source: eLife. 2026 Jun 25;14:RP107748. doi: 10.7554/eLife.107748 (PMC13299597; doi:10.7554/eLife.107748)
Supplement: Peer review history

# Editor report: eLife Assessment

**DOI:** 10.7554/eLife.107748.3.sa0

**Authors:** Sylvia M Evans

**Affiliations:** University of California, San Diego United States

**Keywords:** Solid, Valuable

This is a **valuable** study that presents human single nuclei RNA-seq and spatial transcriptomics data of the developing outflow tract and adult aortic valves that will facilitate research in this area. Data presented are **solid**, with bioinformatics analyses showing cell lineage and trajectory relationships, intriguingly suggesting persistence of embryonic signature in adult aortic valve cells. The latter results would be strengthened by experimental validation.

# Referee report: Reviewer #1 (Public review)

**DOI:** 10.7554/eLife.107748.3.sa1

Summary:

The study by Bobola et al reports single nuclear expression analysis with some supporting spatial expression data of human embryonic and fetal cardiac outflow tracts compared to adult aortic valves. The transcription factor GATA6 is identified as a top regulator of one of the mesenchymal subpopulations and potential interacting factors and downstream target genes are identified bioinformatically. Additional bioinformatic tools are used to describe cell lineage relationships and trajectories for developmental and adult cardiac cell types.

Strengths:

The strengths of the study are studies of human tissue and extensive gene expression data that will be valuable to the field.

Weaknesses:

In the revised manuscript the data remain largely correlative since functional relationships in cell lineages and gene regulatory interactions are based on coexpression data and bioinformatic analyses that were not subjected to further validation.

# Referee report: Reviewer #2 (Public review)

**DOI:** 10.7554/eLife.107748.3.sa2

Summary:

The manuscript by Leshem et al. presents a transcriptomic analysis of the developing human outflow tract (OFT) at embryonic and fetal stages using snRNAseq and spatial transcriptomic. Additionally, the authors analyze transcriptomic data from the adult aortic valve to compare embryonic and adult cell population, aiming to identify persistent embryonic transcriptional signatures in adult cells. A total of 15 clusters were identified from the embryonic and fetal OFT samples, including three mesenchymal and four endothelial clusters. Using SCENIC analysis on the embryonic snRNAseq data, the authors identified GATA6 as a key regulator of valve precursor cells. Spatial transcriptomic analysis of four fetal OFT sections further revealed the spatial distribution of mesenchymal nuclei, smooth muscle cells, and valvular interstitial cells. Trajectory analysis identified two distinct developmental origins of fetal mesenchymal cells: the neural crest and the second heart field. Finally, the authors used snRNAseq data from the adult aortic valve to propose that embryonic transcriptional signatures persist in a subset of adult cells.

Strengths:

(1) The study offers a rich and detailed dataset, combining snRNA-seq and spatial transcriptomics in human embryonic and fetal OFT, which are challenging to obtain.

(2) The use of SCENIC and trajectory analysis adds mechanistic insight into cell lineage and regulatory programs during valve development.

(3) This study confirms GATA6 ss a key regulator of valve precursor cells.

(4) Comparison between embryonic/fetal and adult datasets represents a novel attempt to trace persistence of developmental transcriptional programs.

Weaknesses:

(1) A major limitation is the lack of experimental validation to support key conclusions, particularly the claim of persistent embryonic transcriptional signatures in adult cells.

(2) The manuscript would benefit from a clearer discussion of how these results advance beyond previous studies in human heart and valve development.

(3) The comparison between embryonic and adult data is interesting but would be more convincing with additional evidence supporting the proposed persistence of embryonic transcriptional signatures in adult cells

Comments on revisions:

The final section of the results concludes with the search for a distribution pattern similar to JAG1. The authors end their article by identifying the FOXC1 and OSR1 genes without providing further validation for their discovery, which is regrettable.

# Referee report: Reviewer #3 (Public review)

**DOI:** 10.7554/eLife.107748.3.sa3

Leshem et al have generated a transcriptional cell atlas of the human outflow tract at two developmental timepoints and its adult valvular derivatives. This carefully performed study provides a useful resource for the study of known genes implicated in outflow tract defects and potentially also to discover new disease genes. The authors reveal neural crest and mesodermal contributions to different outflow tract components and show that GATA6, known to play a role in arterial valve development, controls a set of genes expressed in endocardial derived cells during valve development. Interestingly the results reveal intersection with GLI3 and suggest lineage persistence of gene expression through to the adult timepoint, a main new finding of this study.

Comments on revisions:

The authors have carefully addressed previous comments, including the addition of new analysis pointing to potential cooperation between GATA6 and GLI3.

# Author comment: Author response

**DOI:** 10.7554/eLife.107748.3.sa4

**Authors:** Rotem Leshem, Syed Murtuza-Baker, Joshua Mallen, Lu Wang, John Dark, Andrew D Sharrocks, Karen Piper Hanley, Neil Hanley, Magnus Rattray, Simon D Bamforth, Nicoletta Bobola

**Affiliations:** University of Manchester Manchester United Kingdom; University of Manchester Manchester United Kingdom; University of Manchester Manchester United Kingdom; Royal Papworth Hospital NHS Foundation Trust Cambridge United Kingdom; Translational and Clinical Research Institute, Newcastle University Newcastle United Kingdom; University of Manchester Manchester United Kingdom; University of Manchester Manchester United Kingdom; University of Manchester Manchester United Kingdom; University of Manchester Manchester United Kingdom; Newcastle University Newcastle United Kingdom; University of Manchester Manchester United Kingdom

The following is the authors’ response to the original reviews.

**Public Reviews:**

**Reviewer #1 (Public review):**

Summary:

The study by Bobola et al reports single-nucleus expression analysis with some supporting spatial expression data of human embryonic and fetal cardiac outflow tracts compared to adult aortic valves. The transcription factor GATA6 is identified as a top regulator of one of the mesenchymal subpopulations, and potential interacting factors and downstream target genes are identified bioinformatically. Additional bioinformatic tools are used to describe cell lineage relationships and trajectories for developmental and adult cardiac cell types.

Strengths:

The studies of human tissue and extensive gene expression data will be valuable to the field.

Weaknesses:

(1) The expression data are largely confirmatory of previous studies in humans and mice. Thus, it is not clear what novel biological insights are being reported. While there is some novelty and impact in using human tissue, there are extensive existing publications and data sets in this area.

(2) Major conclusions regarding spatial localization, differential gene expression, or cell lineage relationships based on bioinformatic data are not validated in the context of intact tissues.

(3) The conclusions regarding lineage relationships are based on common gene expression in the current study and may not reflect cellular origins or lineage relationships that have previously been reported in genetic mouse models.

(4) An additional limitation is the exclusive examination of adult aortic valve leaflets that represent only a subset of outflow tract derivatives in the mature heart. The conclusion, as stated in the title regarding adult derivatives of the outflow tract, is not accurate based on the limited adult tissue evaluated, exclusive bioinformatic approach, and lack of experimental lineage analysis of cell origins.

**Reviewer #2 (Public review):**

Summary:

The manuscript by Leshem et al. presents a transcriptomic analysis of the developing human outflow tract (OFT) at embryonic and fetal stages using snRNAseq and spatial transcriptomics. Additionally, the authors analyze transcriptomic data from the adult aortic valve to compare embryonic and adult cell populations, aiming to identify persistent embryonic transcriptional signatures in adult cells. A total of 15 clusters were identified from the embryonic and fetal OFT samples, including three mesenchymal and four endothelial clusters. Using SCENIC analysis on the embryonic snRNAseq data, the authors identified GATA6 as a key regulator of valve precursor cells. Spatial transcriptomic analysis of four fetal OFT sections further revealed the spatial distribution of mesenchymal nuclei, smooth muscle cells, and valvular interstitial cells. Trajectory analysis identified two distinct developmental origins of fetal mesenchymal cells: the neural crest and the second heart field. Finally, the authors used snRNAseq data from the adult aortic valve to propose that embryonic transcriptional signatures persist in a subset of adult cells.

Strengths:

(1) The study offers a rich and detailed dataset, combining snRNA-seq and spatial transcriptomics in human embryonic and fetal OFT, which are challenging to obtain.

(2) The use of SCENIC and trajectory analysis adds mechanistic insight into cell lineage and regulatory programs during valve development.

(3) This study confirms GATA6 as a key regulator of valve precursor cells.

(4) Comparison between embryonic/fetal and adult datasets represents a novel attempt to trace persistence of developmental transcriptional programs.

Weaknesses:

(1) A major limitation is the lack of experimental validation to support key conclusions, particularly the claim of persistent embryonic transcriptional signatures in adult cells.

(2) The manuscript would benefit from a clearer discussion of how these results advance beyond previous studies in human heart and valve development.

(3) The comparison between embryonic and adult data is interesting, but would be more convincing with additional evidence supporting the proposed persistence of embryonic transcriptional signatures in adult cells.

**Reviewer #3 (Public review):**

Leshem et al have generated a transcriptional cell atlas of the human outflow tract at two developmental timepoints and its adult valvular derivatives. This carefully performed study provides a useful resource for the study of known genes implicated in outflow tract defects and potentially also for discovering new disease genes. The authors reveal neural crest and mesodermal contributions to different outflow tract components and show that GATA6, known to play a role in arterial valve development, controls a set of genes expressed in endocardium-derived cells during valve development. Interestingly, the results suggest lineage persistence of expression of certain genes through to the adult timepoint, a main new finding of this study.

The following points should be addressed to reinforce the conclusions and emphasize the novel features of this study.

(1) It would be helpful to clarify how these new findings confirm or diverge from what is known from analysis of neural crest and mesodermal lineage contributions to different cell populations in the mouse heart. Did the authors identify any human-specific populations of cells, such as the LGR5 population reported by Sahara et al?

(2) The authors should clarify in the introduction and results that they consider the endocardium to be on the SHF trajectory as indicated in Figure S4C. Please add a reference for this point.

(3) The GATA6 results are interesting and support this experimental approach. The paper would be reinforced if the authors could provide any functional validation (in addition to their GATA6 genomic occupancy data) that the designated target genes are regulated by GATA6. This might involve looking at mutant mouse embryos or cultured cells. Do the authors consider that GATA6 may regulate the endocardial to mesenchymal transition during the early stages of valve development? Or the valve interstitial cell versus fibroblast fate choice?

(4) Do the new findings reveal whether human valves have a direct SHF to VIC trajectory (ie, without transiting through endocardium) as has been recently shown in the murine non-coronary valve leaflet? Relevant to this point, Figure 5E appears to show contributions to a single adult aortic valve leaflet - this should be explained, or corrected.

We sincerely thank the Editor and the Reviewers for their constructive and insightful comments. We have carefully addressed the majority of the points raised and believe the revisions have substantially strengthened the manuscript.

**Recommendations for the authors:**

**Reviewing Editor Comments:**

Overall, the reviewers felt that integrating these datasets with prior snRNAseq datasets on human OFT (de Bono et al, 2025) would enhance analyses and provide broader context.

Several human fetal heart single-cell datasets have been published, including De Bono et al, 2025. We carefully considered whether integrative analyses with these datasets would further strengthen our study. However, there are substantial differences in anatomical scope: most published datasets encompass broad cardiac regions, whereas our study specifically targets the OFT, enabling higher-resolution characterization of OFT-specific cell states. Integration across datasets with markedly different regional compositions would likely be driven by largescale anatomical differences rather than yield additional OFT-specific insight. In addition, cross-study integration requires batch correction. When datasets differ in anatomical scope, as well as developmental timing, and experimental protocols, stronger correction may be needed, increasing the risk of overcorrection and potential loss of biologically meaningful OFTspecific signals.

Importantly, our dataset has been deposited in the Human Cell Atlas and is fully available for future comparative analyses. We therefore believe that broader cross-dataset integration is best undertaken within such harmonized frameworks as more closely matched datasets become available.

Overall, cluster annotations should be more rigorous, which may be facilitated by comparisons with earlier studies.

We have clarified all the points raised by the reviewer regarding cluster annotation. Specifically: (1) the “cardiac” cluster has been renamed “cardiac muscle” to more accurately reflect its transcriptional identity; and (2) we now explicitly state that mesenchymal populations not resolved in the initial global analysis (across all samples) were subsequently defined through dedicated sub clustering analyses performed separately for the adult and developmental datasets. These clarifications have been incorporated into the revised manuscript.

Citation of other spatial transcriptomics studies on human OFT would be useful.

We apologise for missing these contributions. They have now been added to the text.

Can the authors identify a human-specific population of cells, such as the LGR5 population reported by Sahara et al?

While our dataset does not reveal a novel single-gene marker comparable to the human specific LGR5 marker described for the LGR5-positive population by Sahara et al., it does identify a distinct GATA6-enriched embryonic mesenchymal population that functions as a human valve progenitor lineage. Using regulatory network analysis, RNA velocity, lineage tracing and spatial transcriptomics, we show that this GATA6-driven program is specifically associated with semilunar valve morphogenesis and that its transcriptional signature persists in fetal and adult VIC populations. Thus, the novelty of our study lies in defining this human GATA6-regulated valve progenitor population and its lineage trajectory, rather than in the identification of previously unreported single marker genes.

“….Although we have not defined a novel single-gene marker (analogous to LRG5 [Sahara et al]), our identification of a GATA6 network highlights…..”

Further investigation of the specific role of GATA6 would strengthen findings.

FISH studies would indicate whether GATA6 is involved in EMT or fibroblast versus valve interstitial cell fate choice.

We have added a panel to Fig. S2 (D), showing that GATA6 expression is not restricted to specific outflow tract populations. In CS16-17 embryos, GATA6-expressing nuclei are detected across all embryonic clusters. Given this broad expression pattern, FISH analysis would not distinguish whether GATA6 functions in EMT or in fibroblast versus valve interstitial cell fate specification. While we cannot exclude the possibility that GATA6 contributes to EMT, we observe that its expression levels are highest in cluster 4 (post-EMT) cells. This suggests that GATA6 activation is more likely a consequence of the transition rather than its initiating cause (shown in Fig. S2D).

Functional validation of some proposed GATA6 targets would strengthen findings.

To our knowledge, there are currently no publicly available datasets defining the GATA6 regulatory network in human OFT cells or valvular fibroblast progenitors. Existing datasets focus primarily on cardiomyocytes, which arise from a distinct developmental lineage. Given the well-established cell-type and context dependence of transcription factor activity, these datasets are unlikely to provide meaningful insight into regulatory relationships within the valvular lineage examined here.

As noted in the original submission, we previously leveraged published mouse GATA6 ChIPseq data from E11.5 OFT (DOI: https://doi.org/10.7554/eLife.31362) as independent support for the GATA6 regulon identified in our human dataset. In this revised version, we have now extended this analysis by formally quantifying the overlap between the cluster 4 GATA6 regulon and genes bound by GATA6 in the mouse OFT dataset. Using a hypergeometric enrichment test, we found that the observed overlap is approximately two-fold greater than expected by chance and highly significant (p = 1.2 × 10^-33^). This statistical analysis strengthens our original interpretation and provides quantitative support that the identified regulon is strongly enriched for bona fide GATA6-bound targets in a closely related developmental context.

In addition, we examined the spatial expression pattern of the GATA6 regulon gene set and found that it specifically localizes to the semilunar valves (OFT derivatives), consistent with GATA6 activity in this developmental context. This new analysis has been incorporated into Figure 2F of the revised manuscript.

Collectively, the cross-species binding enrichment and valve-specific expression pattern provide orthogonal support for the biological relevance of the identified GATA6 regulon and strengthen the mechanistic interpretation of GATA6 function in OFT and valve development.

As GATA6 has been previously identified in mouse studies, can the authors identify novel transcription factors potentially involved in OFT development?

To identify additional transcription factors potentially involved in OFT development and to define regulators that may confer specificity to GATA6 activity, we compared the GATA6 regulon with the regulons of other cluster 4 transcription factors identified by SCENIC (SOX4, GLI3, RARG, ETV1, GLIS3, BACH2, ZNF423, FOXO3, ZBTB20).

While all cluster 4 regulators share some downstream targets, GLI3 regulon showed approximately twice the degree of overlap with the GATA6 regulon compared to the other factors. This suggests a potential functional interaction between GATA6 and GLI3 in OFT associated mesenchyme. Consistent with this, cooperation between GATA6 and GLI3 has been reported in mouse limb development. These findings have now been incorporated into the Results section, and co-expression of GATA6 and GLI3 in CS16-17 populations is shown in Figure S2DE.

Although GATA6 has previously been implicated in OFT development, SCENIC analysis provides mechanistic insight by defining the downstream gene programs active in specific human embryonic lineages. Thus, the novelty of our findings lies not in re-identifying GATA6, but in characterizing its regulon in human OFT- and valve-associated mesenchyme and identifying potential cooperating regulators such as GLI3.

Embryonic signatures in adult valve cells are an interesting finding, that should be further explored by pseudotime trajectories, which may also indicate whether SHF cells have a direct trajectory to VIC (without transiting endocardium), as recently shown in mice.

We included all embryonic populations, including cardiac progenitor cells (SHF), in the pseudotime trajectory analysis. However, we did not observe evidence of a direct trajectory from SHF cells toward VIC. In contrast, the same analysis consistently identified a trajectory linking endocardial cells to VIC, supporting an endocardial origin in our dataset.

**Reviewer #1 (Recommendations for the authors):**

(1) Major conclusions regarding cell lineages and derivatives are based on common gene expression patterns and bioinformatic tools. Thus, these conclusions are not based on empirical data, and assumptions regarding lineages based on gene expression may not be accurate. The language related to lineage analysis, derivative, and longitudinal gene expression is not supported by data. For example, studies in mice have shown that aortic valve interstitial cells from endocardial cushions and neural crest-derived lineages have overlapping patterns of ECM gene expression and cannot be easily distinguished in adults. Thus, it is not possible to determine derivation and cell origins based on gene expression alone.

While we fully acknowledge that gene expression-based analyses provide correlative rather than direct lineage-tracing evidence, the Reviewer’s statement that “it is not possible to determine derivation and cell origins based on gene expression alone,” and the example cited in support, appear to equate global transcriptional similarity with the distinct embryonic transcriptional signatures that underpin our analysis.

As the Reviewer notes, a given differentiated cell type can derive from different embryonic progenitors. Due to functional convergence, differentiated cells often exhibit highly similar expression profiles that reflect their shared function rather than developmental origin. Consequently, discriminating embryonic origins based on global expression profiles, or even for highly distinctive genes of differentiated cells, is very challenging. The example cited by the Reviewer - overlapping ECM gene expression in aortic valve interstitial cells derived from endocardial cushions and neural crest - illustrates precisely this point.

However, our analysis does not rely on global transcriptional similarity or on markers of mature differentiated cells. Instead, we specifically identified gene sets that are highly distinctive of embryonic clusters prior to the onset of differentiation. These signatures are enriched for transcription factors and signaling molecules that define developmental identity, rather than functional effector genes associated with mature cell states. We have shown that these embryonic signatures persist in fetal cells (which already express differentiated markers but are developmentally closer to the embryonic stage relative to adult cells) and remain detectable, albeit attenuated, in adult cells. It is these distinctive embryonic transcriptional signatures, rather than global or shared functional gene expression, that we have used to infer potential lineage relationships.

We fully acknowledge that this constitutes correlative evidence rather than direct lineage tracing, which is not feasible in human studies. However, the persistence of embryonic regulatory signatures into fetal and adult stages provides a biologically plausible link to developmental origin. This persistence most plausibly reflects partial retention of ancestral embryonic transcriptional programs in descendant cells, rather than de novo activation later in life of embryonic genes that were never previously expressed in that cell’s lineage.

(2) Most of the findings related to cell composition, gene expression, and cell lineages seem to be largely confirmatory of previous reports. Novel findings should be emphasized and validated in the tissues.

We agree that several aspects of our dataset reproduce and extend findings from previous human and animal studies, which we regard as an important validation of the atlas. However, our study also provides multiple novel insights that are directly supported by our spatial data. Specifically, we (i) identify a GATA6-enriched embryonic mesenchymal valve progenitor population, (ii) delineate its GATA6 transcriptional regulon and direct targets implicated in OFT and valve disease, and (iii) trace its embryonic transcriptional signature into fetal and adult valve interstitial cell populations. These findings are strengthened by our spatial transcriptomic data, which maps the GATA6 regulon and key targets to the semilunar valves and adjacent arterial root, providing in situ validation of both cell identity and gene expression patterns (see Fig. 3 and the newly added Fig. 2F). We have revised the Discussion to more explicitly highlight these novel aspects and their spatial validation in the final

“In summary, our work goes beyond confirming previously reported cell types by (i) defining a GATA6-regulated human valve progenitor lineage and its descendants, (ii) establishing distinct embryonic origins for smooth muscle and valvular fibroblasts, and (iii) demonstrating persistence of embryonic signatures in adult valve cell populations. These findings are directly supported in tissue by our spatial transcriptomics data, which map these lineages and regulatory programs to defined anatomical domains within the human OFT and semilunar valves.”

(3) The developing outflow tract of the heart contributes to more than just the aortic valve leaflets in adults. Additional conotruncal structures need to be evaluated in order to define adult derivatives of the developing outflow tract as described in the title.

The title has been changed to reflect that only adult aortic valves were examined.

(4) Major conclusions regarding the GATA6 regulatory network and downstream target genes are not validated in the context of the developing outflow tract or adult valves. Is GATA6 expression restricted to specific outflow tract populations? Is GATA6 binding or responsive gene expression detected for the indicated target genes?

We performed additional analyses that further reinforce the relationship between GATA6 and its target genes and support the biological relevance of GATA6 downstream targets in arterial valve development. Below, we address the specific questions raised by the reviewer.

(1) Is GATA6 expression restricted to specific outflow tract populations?

GATA6 expression is not restricted to specific outflow tract populations. In CS16-17 embryos, GATA6-expressing cells are detected across all embryonic clusters; however, expression levels are highest in cluster 4 (valve precursor cells).

Despite this broad expression pattern, SCENIC identifies GATA6 activity (i.e., a GATA6 regulon) specifically in cluster 4. This apparent restriction of GATA6 regulatory activity to cluster 4 may be explained, at least in part, by its elevated expression levels within this cluster. Alternatively, given that transcription factors often act in a combinatorial manner, GATA6 may co-regulate its target genes in cluster 4 together with additional cluster-specific regulators. To explore this possibility, we compared the GATA6 regulon with the regulons of other cluster 4 transcription factors identified by SCENIC (namely SOX4, GLI3, RARG, ETV1, GLIS3, BACH2, ZNF423, FOXO3, ZBTB20) in order to identify potential co-regulatory modules. As expected, since these regulons are sampled from the subset of genes enriched in cluster 4, all regulators share a substantial proportion of downstream targets with GATA6. However, GLI3 stands out, showing approximately twice the degree of overlap compared to the other factors. This suggests a functional interaction between GATA6 and GLI3, consistent with previously reported cooperation in mouse limb development. These results have been incorporated into the Results section, and the expression of GATA6 and GLI3 in CS16-17 cell populations is shown in Fig. S2DE.

(2) Is GATA6 binding or responsive gene expression detected for the indicated target genes?

We were unable to find public data describing the GATA6 regulatory network or its downstream targets in the specific human cell types examined here (OFT cells; valvular fibroblast progenitors). Available datasets focus primarily on cardiomyocytes, which arise from a distinct lineage, and because transcription factor function is highly cell-type and context dependent, these datasets are unlikely to be helpful in inferring regulatory relationships in the valvular lineage.

The strongest validation for the GATA6 regulon identified in this study comes from the mouse GATA6 occupancy data (this was included in the original manuscript). Although derived from a different species, GATA6 binding has been profiled in a highly related developmental context, the OFT. To assess the relevance of these data to our human findings, we performed a hypergeometric test comparing the GATA6 regulon identified in cluster 4 (this study) with genes bound by GATA6 in E11.5 mouse OFT ChIP-seq data (DOI: https://doi.org/10.7554/eLife.31362). The observed overlap is substantially greater than expected by chance: it is approximately twice the expected value, and the enrichment is highly significant (p = 1.2 × 10^-33^). Biologically, this strongly supports the interpretation that many genes within GATA6 regulon are likely to be direct GATA6 targets, or at minimum are strongly associated with GATA6 binding, rather than representing a random gene set. This analysis has been added to the revised manuscript.

In this revised version of the manuscript, we also overlapped the expression of GATA6 regulon genes to our fetal spatial transcriptomics data. GATA6 regulon was identified in embryonic cluster 4, whose expected trajectory is fetal valvular fibroblasts (cluster 12). Remarkably, GATA6 regulon genes are expressed in both the aortic and pulmonary valves, and their expression pattern aligns closely with HAPLN1-positive valvular fibroblasts (cluster 12), further supporting the biological relevance of this gene set. This new data has been added to Fig 2(F).

Together, the strong enrichment of GATA6 regulon genes among GATA6-bound targets in the OFT, and the specific expression of this gene set within the arterial valves (cluster 4 descendant cells), support the biological relevance of GATA6 downstream targets in arterial valve development and disease. In addition, we identify GLI3 as a potential GATA6 co-binding partner.

(5) What are "cardiac" cell types in the embryonic single cell clustering? Are these cardiomyocytes? Cardiac is an ambiguous term if the cells being analyzed are all in the heart.

Thank you for highlighting this ambiguity. The “cardiac” population refers specifically to cardiac muscle cells. We have updated the labels in Fig. 1E, 1F, and Fig. S3A to make this explicit.

(6) The methods and analytical tools seem fairly standard for single nuclear gene expression and spatial genomics studies. What are the new tools and resources being reported? The "novel lineage tracing algorithm" mentioned in the methods is not well described. A Cellxgene VIP app is mentioned, but is not described in detail. Also, it seems to be housed on a local server, which is not optimal.

The description of the lineage tracing algorithm has been expanded in the method’s section of the paper.

The data has been submitted to the Human Cell Atlas, a coordinated global effort to systematically map human cell types using standardized, interoperable formats. Public access via cell x gene enables interactive visualization, gene-level queries, and cross-dataset comparisons without requiring advanced computational expertise. This broad accessibility enhances reproducibility, facilitates integration with complementary single-cell and spatial datasets, and maximizes the visibility, transparency, and long-term impact of our work.

(7) Only adult aortic valves from females were included in the study.

The rationale for using female tissues has been explained in the result section:

We collected female samples to mitigate individual variability and maximise the possibility to analyse healthy aortic valves, justified by the lower incidence and severity of aortic disease in females versus males.

(8) In many of the figures, the font size of the text is too small to read.

We have increased the font size in all figures where this was compatible with the layout. For the larger plots, additional enlargement would necessitate scaling the panels beyond the allowable page dimensions, and therefore could not be implemented.

(9) "CAT" is not a commonly used abbreviation for congenital heart anomalies related to persistent truncus arteriosus.

CAT is now the preferred term for PTA as latinised terms are no longer used.

**Reviewer #2 (Recommendations for the authors):**

Overall, this study is thoughtfully conducted and offers valuable observations that contribute to our understanding of valve morphogenesis. However, my main concern is the lack of experimental validation to support the findings, particularly the conclusion regarding the persistence of transcriptional signatures in adult cells, which is not sufficiently substantiated or clearly argued. It is unclear how this study advances beyond previous research in humans.

Major points:

(1) Several recent studies have applied spatial transcriptomics to human embryonic and fetal hearts, including OFT (Asp et al., 2019; Queen et al., 2023; Farah et al., 2024; De Bono et al., 2025). It is disappointing that the authors did not acknowledge these important contributions.

We apologise for missing these contributions. They have now been added to the text.

(2) The present study used snRNAseq to explore the transcriptional signature of the fetal OFT. A similar approach was used by De Bono et al. (2025) to analyze fetal hearts. Integrating these complementary snRNAseq datasets could enhance the current analysis and provide broader context for the findings.

The reviewers suggested that integrating our datasets with prior snRNA-seq datasets on human OFT (de Bono et al., 2025) could enhance the analyses and provide broader context. While several fetal heart datasets have been published (e.g., Sahara et al.), our study focuses specifically on the OFT. These other studies do not perform cross-dataset comparisons. We therefore do not see a strong rationale for integrating ours, especially given that those datasets cover much larger regions of the heart.

(3) Figure 1 presents 18 distinct clusters identified through unsupervised clustering. The authors classify three of these clusters broadly as mesenchymal cells. However, the term "mesenchymal cells" lacks precision. The authors should clarify why these clusters were not more specifically defined as fibroblasts or myofibroblasts based on marker expression.

Clustering of the full dataset does not provide sufficient resolution to distinguish all mesenchymal cell types. The clusters broadly annotated as mesenchymal comprise heterogeneous populations, including both undifferentiated embryonic mesenchymal cells and more differentiated fetal mesenchymal cells. These mesenchymal clusters were therefore further subclustered, and the resulting cell identities are described in detail in the Results sections corresponding to Fig. 2 and Fig. 3.

(4) The authors used SCENIC on their snRNAseq datasets to infer key cell fate regulators and identified GATA6 as a top regulator of embryonic mesenchymal cluster 4. However, the rationale for focusing on GATA6, which is already known to be associated with CHD in humans, is not fully convincing. Why not investigate a transcription factor whose role in valve development remains unexplored?

There are two key outcomes from a SCENIC analysis: (1) the identification of major transcriptional regulators driving the differentiation of a given cluster, and (2) the identification of their regulons (the downstream gene programs they control). While GATA6 is indeed already known to be associated with CHD in humans, including valve malformations and major OFT defects, its downstream targets in the relevant human developmental lineages have not been defined. Understanding these targets is essential for clarifying the molecular basis of GATA6-mediated CHD. Thus, the significance of our result does not lie in the rediscovery of GATA6 as a CHD-related factor, but in identifying the genes it regulates in embryonic OFT- and valve-associated mesenchyme. These GATA6-controlled genes in the OFT and valves represent biologically plausible candidate genes for human OFT defects, as disruption of GATA6 targets could similarly contribute to CHD.

In this revised version we have performed a hypergeometric test showing that GATA6 regulon genes are significantly enriched among genes bound by GATA6 in the OFT. Biologically, this strongly supports the interpretation that many genes within the GATA6 regulon are likely to be direct GATA6 targets, or at minimum are strongly associated with GATA6 binding in the OFT, rather than representing a random gene set.

We have also mapped the expression of GATA6 regulon to the semilunar valves. Collectively, these analyses demonstrate that the GATA6 regulon captures a biologically coherent and developmentally relevant program, offering new mechanistic insight into how GATA6 influences OFT and valve formation and how its disruption may contribute to CHD.

(5) Several studies have already suggested a role for GATA6 in EMT. Do the authors propose that GATA6 regulates this process during embryonic valve development? Once again, validation using FISH would be important to support these findings.

We do not propose that GATA6 directly regulates EMT during embryonic valve development. We rather make two independent observations: (1) cluster 4 derives from cluster 7 (likely through EMT); (2) GATA6 regulates cluster4-specific genes.

The first observation is supported by RNA velocity, which links cluster 7 to cluster 4. Supporting this interpretation, endothelial cluster 7 is enriched for genes associated with arterial valve development, and mesenchymal cluster 4 cells are identified as progenitors of fetal valve fibroblasts. Because cluster 7 is endothelial and cluster 4 is mesenchymal, this trajectory suggests an endothelial-to-mesenchymal transition.

Second, SCENIC analysis identifies GATA6 as a regulator of cluster 4 genes. Additionally, the GATA6 regulon shows distinct localization to the formed valves in fetal cells (new data added to Fig 2F). Together these findings support the notion that GATA6 regulates a gene program specific to the cell populations that will give rise to the valves and that these genes remain selectively expressed in valve cells once the arterial valves have formed.

While we cannot exclude the possibility that GATA6 contributes to EMT, we observe that GATA6 expression levels are highest in cluster 4 (post-EMT) cells, suggesting that its activation may be a consequence of the transition rather than its initiating cause (now shown in Fig S2D).

For validation using FISH, please see response to point 6 below

(6) I found it curious that the ST section was used to validate MECOM expression (Figure 2I), while ST had not yet been introduced at this point in the manuscript. Validation using FISH would have been a more appropriate approach.

Thank you for drawing attention to this discrepancy. Spatial transcriptomics is now introduced before MECOM analysis, in the Results section pertaining to Figure 2F

“…spatial transcriptomic analysis of a later stage (12pcw) OFT shows that GATA6 regulon is mainly restricted to the aortic and pulmonary valves (Fig 2F)”.

With regard to this and the above comment concerning FISH, while RNA FISH/RNAscope would provide an additional orthogonal approach, the Visium-based spatial transcriptomics platform directly measures MECOM transcripts in tissue sections and, in our view, represents an appropriate and sufficiently sensitive method for validating its spatial distribution in the human OFT. We have therefore relied on the spatial transcriptomics dataset to confirm and validate gene expression patterns, rather than performing additional FISH experiments. We now explicitly state that this approach serves as an independent in situ validation of gene expression, including MECOM.

(7) "Spatial resolution of mesenchymal nuclei in the OFT" section: It is unclear which cluster the authors are referring to in this section.

As mentioned in the text, we “mapped the five fetal mesenchymal clusters to distinct structures in the OFT” and used distinctive markers to confirm spatial assignments.

(8) The authors should justify their choice to use Cell2location instead of a deconvolution method.

We selected cell2location because it provides a probabilistic, hierarchical Bayesian framework that explicitly models technical variability across both single-cell reference data and spatial transcriptomics platforms. Rather than relying on predefined marker genes or simple linear regression, cell2location leverages the full transcriptomic profile of reference single-cell data and incorporates a factor analysis-based framework to model shared transcriptional signatures and latent structure across cell types. This approach improves discrimination between closely related cell states and reduces sensitivity to gene selection bias. Additionally, the probabilistic formulation yields uncertainty estimates for inferred cell abundances, enhancing interpretability and statistical rigor. Together, these features make cell2location particularly well suited for resolving complex cellular composition in our fetal human tissue spatial transcriptomics data.

(9) Figure 3: Cluster 9 is identified as endothelial, yet it includes markers such as MYH11 among its top genes, a gene more commonly associated with cells at the base of the aorta. This raises questions about the accuracy of the cluster annotation.

We could not find the definition of cluster 9 as endothelial to which the reviewer refers to. In Fig 3, both in the result text and in the figure legend, cluster 9 is identified as smooth muscle, which is consistent with *MYH11* expression. The endothelial cluster is shown in Fig S3C.

(10) The approach used to trace embryonic signatures in adult cells, based on overlap with the top 100 genes in embryonic clusters, relies largely on gene expression similarity, without incorporating lineage inference tools such as RNA velocity or pseudotime analysis. This limits the ability to distinguish true developmental relationships from shared functional programs. I believe that the use of aggregated adult samples may mask individual variability. Validation in separate samples (AV1 and AV3) lacks statistical rigor. The observed lower expression of embryonic genes in adult cells further complicates interpretation, raising the possibility that these signatures reflect residual expression rather than persistent lineage markers.

We thank the reviewer for the opportunity to clarify our approach.

We fully agree that tools such as RNA velocity and pseudotime are powerful for capturing short-term dynamic transcriptional changes and inferring lineage trajectories within continuous developmental processes. Indeed, we applied RNA velocity and identified a transition between clusters 7 and 4 in embryonic cells (Fig 2). However, as noted in the Results section, “trajectory inference methods failed to establish lineage relationships between embryonic and fetal populations”. These methods assume temporal continuity and comparable transcriptional kinetics between cells. When comparing samples separated by large developmental intervals (e.g., embryonic versus adult tissues), these assumptions do not hold: RNA velocity vectors become unreliable and may even yield biologically meaningless directions. Therefore, rather than forcing a continuous trajectory across temporally distant datasets, we employed an anchoring approach designed to identify conserved transcriptional programs and potential lineage correspondences between embryonic and adult cell types.

To address the concern about individual variability, we performed analyses both on aggregated adult samples and on individual replicates (AV1 and AV3). The results were highly consistent across both levels of analysis, and statistical significance was supported by very low p-values, indicating that the observed patterns are robust and reproducible. We therefore believe our analysis in independent samples is statistically sound.

Finally, we agree that adult cells display lower expression of embryonic genes, and we acknowledge that these signatures may represent residual rather than persistent expression. This observation aligns with our intended interpretation: our goal was not to demonstrate enduring embryonic marker expression, but to highlight that adult cells retain transcriptional traces that connect them to their developmental origins.

**Reviewer #3 (Recommendations for the authors):**

(1) Please clarify if MEIS1, JAG1, ROR1, PRDM6 have been previously implicated in neural crest cell development. Are these then new potential regulators of neural crest cells? The same applies to SOX6 for the mesodermal population.

The main reason for selecting these genes (MEIS1, JAG1, ROR1, and PRDM6 in cluster 20, and SOX6 in cluster 4) is that they serve as distinctive markers of specific embryonic clusters. Because their expression remains restricted at later developmental stages, they allow reliable tracing of bona fide descendant cells originating from cluster 20 and cluster 4 into fetal and adult tissues. Importantly, MEIS1, JAG1, ROR1, and PRDM6 were not chosen as new potential regulators of neural crest (NC) cells, but rather because their expression is enriched in cluster 20 and remains restricted at later developmental stages, allowing reliable tracing of bona fide descendant cells originating from cluster 20. Since cluster 20 is, based on transcriptional profiles, the embryonic mesenchymal cluster most closely related to the NC lineage, these markers enable lineage tracing of NC-descendent cells. Nonetheless, these genes have all been linked to neural crest biology, either through known functional roles or through specific expression patterns associated with NC development.

Similarly, SOX6 was selected for its restricted expression in cluster 4, a pattern that is preserved in its descendant populations, making it a suitable marker for tracking the mesoderm-derived lineage.

(2) Please comment in the text whether any regional transcriptional differences (rather than cell type differences) were detected between the aortic and pulmonary regions.

We have added the following text to the result section related to Fig 3: “No molecular differences or distinguishing markers were identified between the aortic and pulmonary valves.”

(3) There appear to be no myocardial cells in the adult valve tissue - the authors could discuss what the fate of myocardium is in the embryonic OFT. Are they only looking at a subset of derivatives of the embryonic OFT?

Our adult dataset represents the aortic valve complex and adjacent arterial root tissue (a subset of outflow tract derivatives) rather than the entire outflow tract (this has now been specified in the title). Spatial transcriptomic analysis identified myocardial gene expression within the ventricular and outflow tract walls at CS16-19, but not within the valve leaflet cluster (Queen et al., 2023). This is consistent with previous observations that myocardium contributes to the arterial root and supports early cushion formation, but does not persist in mature valve tissue, which becomes predominantly fibrous and populated by valve interstitial cells. This explanation has been added to the analysis of cell populations in the valves.

(4) Please equate Carnegie stages 13-23 to embryonic days or weeks of gestation in the first paragraph to help the general reader.

We have added the suggested clarification and noted that this period spans four weeks of human development, rather than the three weeks previously indicated. The text has been updated accordingly.

(5) I suggest rewriting the first sentence of the introduction using the plural, as there are many different types of CHD.

The sentence has been changed accordingly.

(6) It would be helpful to add the persistence of embryonic signatures into adult valve cell types in Figure 4E.

We thank the reviewer for this helpful suggestion. To address this point, we have now added an analysis of the persistence of embryonic signatures in adult valve cell types to Figure 4E. Specifically, we selected 10 representative genes from the 100-gene embryonic signature lists of cluster 4 and cluster 20 and projected their expression onto the t-SNE shown in Figure 4E. The combined (module) expression of these 10 genes is now shown in Figure S6E, and the expression of the individual genes is presented in the newly added Figure S7.

We would like to clarify that our statistical framework identifies potential descendant populations based on significant enrichment of an embryonic gene signature. Therefore, individual embryonic genes are not necessarily expected to be expressed exclusively or uniformly within a single adult population.

(7) Please explain how the 2-dimensional plot in 2J relates to the other plots.

The plot originally shown in Fig 2J (now Fig 2K) was generated by applying RNA velocity exclusively to CS16-17 nuclei. Developmental nuclei (excluding adult samples) were subclustered as shown in Fig S2AB, resulting in the 5 clusters of embryonic nuclei analysed in Fig 2J: cardiac muscle (2, 17), endothelial (7), and mesenchymal (4, 20).
